# Platelet hyperactivity and fibrin clot structure in transient ischemic attack individuals in the presence of metabolic syndrome: a microscopy and thromboelastography^®^ study

**DOI:** 10.1186/s12933-015-0249-5

**Published:** 2015-07-04

**Authors:** Mia-Jeanne van Rooy, Wiebren Duim, Rene Ehlers, Antoinette V. Buys, Etheresia Pretorius

**Affiliations:** Department of Physiology, Faculty of Health Sciences, University of Pretoria, Private Bag x323, Arcadia, 0007 South Africa; Department of Neurology, Faculty of Health Sciences, University of Pretoria, Arcadia, 0007 South Africa; Department of Statistics, Faculty of Natural Sciences, University of Pretoria, Arcadia, 0007 South Africa; Unit of Microscopy and Microanalysis, Faculty of Natural Sciences, Arcadia, 0007 South Africa

**Keywords:** Transient ischemic attack, Coagulation, Platelets, Fibrin network, Metabolic syndrome, Chronic inflammation, Electron microscopy, Thromboelastography^®^

## Abstract

**Background:**

Strokes are commonly preceded by transient ischemic attacks (TIAs). TIA is often associated with metabolic syndrome (causing chronic inflammation), resulting in a proinflammatory- and procoagulant-environment. The aim of this study was to determine whether platelet- and fibrin network-morphology or coagulation profiles of individuals that suffered a TIA in the presence of metabolic syndrome was altered when compared to healthy individuals.

**Materials and methods:**

The study consisted of 40 voluntary participants. Twenty individuals that suffered a TIA in the previous 48 h with at least two metabolic syndrome risk factors present and twenty healthy age-matched controls. Scanning electron- and atomic force microscopy was used to study platelet- and fibrin-morphology, atomic force microscopy was used to study platelet- and fibrin fiber-elasticity and thromboelastography^®^ for the study of coagulation profiles. Statistical analysis was performed to compare the two groups. In all cases a *p*-value of less than 0.05 was considered statistically significant.

**Results:**

Platelets of the control group appeared spherical with few pseudopodia present while the platelets of the TIA individuals presented with numerous pseudopodia and spreading, indicating activation. Platelet aggregation was also present. The fibrin networks of the healthy individuals consist of thick and thin fibers that form an organized network of fibers. The fibrin networks of the TIA individuals appeared less organized with less taut fibers. Fibrin fiber thickness was found to be significantly increased in the TIA group (*p*-value <0.001) when compared to healthy controls. The thicker fibers formed irregular networks with thick masses of fibrin fibers. Platelet and fibrin fiber elasticity was found to be significantly lower in the experimental group (*p*-value 0.0042 and *p*-value 0.0007 respectively). The hemostatic profiles of the diseased individuals did not differ significantly (*p*-value > 0.05) from the healthy controls, indicating a normal functioning coagulation cascade.

**Conclusion:**

The findings indicate that pathological clot formation is not caused by alterations in the coagulation cascade but rather by the premature activation of platelets (as a result of chronic inflammation) that in turn causes altered fibrin formation.

## Background

Ischemic heart disease and stroke accounts for 25 % of deaths in the world every year and in the USA 240 000 people suffer a transient ischemic attack (TIA) annually [1, 2]. TIA is defined as: “*A brief episode of neurologic dysfunction caused by focal brain or retinal ischemia, with clinical symptoms typically lasting less than one hour and without evidence of acute infarction*” [3, 4]. TIAs, similar to angina, is usually an ominous predictor of future vascular complications [5]. The likelihood of suffering a stroke after a TIA is higher than suffering a recurrent stroke after a first stroke, indicating that TIAs should be treated with the utmost care in order to avoid possible events that may have severe neurological consequences [5]. Although several physical characteristics (such as age) can increase the likelihood of an ischemic event occurring [1], metabolic syndrome is currently the most prominent risk factor associated with these events [6]. For this manuscript the National Cholesterol Education Program – Adult Treatment Panel III (NCEP ATP III) definition was employed since this definition links the pro-thrombotic- and proinflammatory-state seen with metabolic syndrome and thrombotic events. Furthermore since no definite conclusion has been reached regarding criteria that should be absolutely required, this definition circumvents this controversy by suggesting that all risk factors play a role in the development of metabolic syndrome and its complications [7].

Metabolic syndrome is linked to chronic inflammation - another major risk factor for the development of ischemic events, since inflammation is linked to alterations in coagulation [6, 8]. For a review on blood coagulation in normal physiology and specifically platelet action see one of the following [6, 9–11]. Important to mention for this study is that fibrin and platelets are the major role players in both the elasticity and rigidity of the formed clot [12]. The elastic properties of fibrin and platelets allow clot deformation to occur under normal physiological conditions, but also influence the rigidity of the clot. Since fibrin fibers are stiffer when stretching than when bending, the elasticity of the clot is dependent on the cross-linking of the fibers as well as the interaction between the fibers and platelets [13, 14]. Once fibrin fibers and platelets have formed the initial clot, platelet contraction occurs that decreases the size of the clot (retraction) and also places strain on the fibrin fibers, thereby altering fibrin fiber rigidity as well as organisation of the fibers [12].

In the presence of inflammation, normal physiological coagulation is altered when the inflammatory mediators modify the endothelium in the damaged area to become proinflammatory and pro-thrombotic [9]. Importantly inflammation is further associated with thrombosis since coagulation is stimulated by inflammation and inflammation is enhanced in the presence of procoagulant activity [15–17]. Inflammation influences coagulation by increasing the production of coagulation proteins, reducing the activity of the anticoagulant pathway and by preventing fibrinolysis [17, 18]. Together these alterations could lead to the formation of pathological thrombi resulting in infarcts in the heart or brain. Although atherosclerosis may be one cause of TIA, the presence of metabolic syndrome could affect the coagulation system in some way, leading to a TIA even before atherosclerosis develops.

Since a large number of individuals that are affected by metabolic syndrome suffer cerebral ischemic events, finding new therapeutic targets by elucidating new factors that contribute to these incidents are crucial to the treatment and prevention of the events. This manuscript consequently focuses on the alterations of the coagulation system in TIA individuals in the presence of the metabolic syndrome. Special attention was paid to platelets and fibrin networks and the effect on the overall hemostatic profile of the participants using scanning electron- (SEM) and atomic force-microcopy (AFM) as well as thromboelastography^®^ (TEG^®^).

## Materials and methods

### Sample selection

Twenty voluntary participants (between the ages of 39 and 78) that suffered a TIA in the presence of the risk factors associated with metabolic syndrome using the NCEP ATP III criteria were included in this study as experimental group, along with 20 healthy, age-matched individuals that served as the control group. The Human Ethics Committee of the University of Pretoria granted ethical clearance for this study. A qualified neurologist made the diagnosis of TIA after several diagnostic (e.g. magnetic resonance imaging) and laboratory-based blood tests (e.g. platelet count and coagulation parameters) were performed. Blood samples, via venipuncture, for this study were collected after informed consent was obtained from all participants. Blood was drawn from patients within 48 h after the TIA attack to ensure that the acute phase of the attack was studied. Several inclusion and exclusion criteria were used to determine eligibility for this study.

#### Experimental group

*Inclusion criteria:*

- A definite diagnosis of TIA (any uncertainty regarding diagnosis resulted in ineligibility for participation in the study)
- Males and females over the age of 18 years
- At least two metabolic syndrome symptoms present when the TIA occurred

*Exclusion criteria:*

- Smoking
- Heavy drinking as indicated in patient history that could confound inflammatory effects
- Hormone replacement therapy
- Known common inflammatory conditions such as asthma, human immunodeficiency virus (HIV) or tuberculosis not related to the risk factors associated with metabolic syndrome
- Treatment with tissue plasminogen activator directly after the event to prevent any confounding of results regarding the coagulation profile

Since metabolic syndrome is a pre-diabetic condition, diabetes was not excluded in this study.

Of the 20 experimental participants all 20 were viscerally obese, 17 had diagnosed hypertension, 18 hypercholesterolemia and 12 a high fasting glucose. No carotid stenosis was detected in any of the participants with magnetic resonance angiography or with echocardiography of the carotid arteries. Two individuals had subclinical calcified plaque present in the carotid arteries, 2 had an increased intima media thickness unilaterally and 2 bilaterally. Only 1 patient was diagnosed with atherosclerosis. Two individuals had valve disease, most likely as a result of hypertension, 1 had left ventricular hypertrophy and 3 patients tachycardia. Although tachycardia had been diagnosed at the time of the attack the electrocardiogram (ECG) of 1 patient indicated tachycardia, the other 2 had normal ECG recordings due to effective treatment. A careful inventory of chronic medication or treatments after the attack was taken to determine whether the medication could affect the results of this study. The medications with known effects on coagulation are summarized in Table 1. Since all the patients were either on anticoagulants prior to the attack or treated with anticoagulants and/or antiplatelet medication after the attack, these drugs could decrease the coagulation potential of the individual. Since the findings of this study could be blunted by the presence of the drugs, the significant findings of the study regardless of the presence of anticoagulants or antiplatelet therapy makes these findings even more important. For this reason the use of anticoagulants or antiplatelet medication was not used as an exclusion criterion in this study.

**Table 1:** Medication and the effect on coagulation

Medication	Nr of patients	Medication administered	Comment	Ref
Anticoagulant	4	Warfarin, Xarelto, Clexane	Decrease production of coagulation factors by the liver.	[41]
Cox-1 inhibitor	12	Ecotrin, Disprin CV, Disprin	Inhibits cox-1 activity thereby preventing the production of TXA_2_ and platelet aggregation	[42]
P2Y_12_-inhibitor	5	Plavix (Clopidogrel)	Prevents release of ADP and thereby the activation of platelets and subsequent platelet aggregation	[43]
β-blocker	6	Adco-biscor, Bilocor, Tenbloka, Bisoprolol, Hypotone, Carloc	Down regulates TXA_2_ production, but not platelet aggregation and therefore not significant effect on coagulation	[43]
Decreased fasting glucose agent	4	Arrow metformin, Glucophage, Galvus, Starlix	Decreases the amount of PAI-1 available, thereby influencing fibrinolysis	[44]
Synthetic insulin	1	Levemir	Hyperinsulinaemia may decrease fibrinolysis, so treatment can normalise fibrinolytic activity	[45]
GABA derivatives	2	Nootropil	Appears to have an antithrombotic effect, but mechanism not elucidated as yet	[46]

*Nr* Number, *Ref* Reference, *Cox-1* cyclo-oxygenase 1, *β-blocker* Beta blocker, *TXA2* Thromboxane A2, *GABA* Gamma-aminobutyric acid

#### Healthy group

*Inclusion criteria:*

- Over the age of 18 years
- No inflammatory conditions

*Exclusion criteria:*

- Smoking
- Heavy drinking
- Chronic diseases or medication use
- Hormone replacement therapy or other medication that could affect coagulation
- Any risk factors associated with metabolic syndrome

### Scanning electron microscopy

SEM was used to study platelet- and fibrin fiber-morphology as well as to determine fibrin fiber thickness in both groups. Platelet- and fibrin network-morphology were studied by preparing platelet-rich plasma (PRP). PRP was prepared by drawing 5 ml of blood in a sodium citrate vacuum tube (3,8 % final concentration: BD Vacutainer). The blood sample was centrifuged at 3600 × g for 2 min. All SEM samples were prepared on 10 mm glass coverslips.

Ten μl of PRP was used to study platelet morphology and 10 μl of PRP was mixed with 5 μl of human thrombin to study fibrin network morphology. Since red blood cells that are incorporated into clots have been found to alter the thickness of fibrin fibers, PRP was used to study fibrin thickness. The South African National Blood Service (SANBS) supplied human thrombin. The thrombin solution was at a concentration of 20 U/ml and was made up in a biological buffer containing 0.2 % human serum albumin. When thrombin is added to the freshly prepared human PRP or whole blood, the fibrinogen is converted to fibrin and other intracellular platelet components are released into the coagulum. These components include, but are not limited to transforming growth factor, platelet-derived growth factor and fibroblastic growth factor. The glass coverslips were incubated at 37 °C for 10 min. After incubation the cover slips were placed in phosphate buffer (PB) solution on a shaker and washed for 20 min. The wash step was included to remove any blood proteins that may be entangled in the formed blood clot. The samples were then fixed for 30 min in a mixture of 2,5 % gluteraldehyde and 2,5 % formaldehyde solution. After the fixation step the samples were washed three times in PB solution for 3 min to remove any residual fixative. The samples were then fixated a second time in 1 % osmium tetroxide (OsO_4_) for 15 min. This step was followed by another washing step, which included three washes for 3 min in PBS. The samples were then serially dehydrated in 30 %, 50 %, 70 %, 90 % and finally three times in 100 % ethanol. The samples were dried using hexamethyldisilazane (HMDS), and mounted and coated with carbon. Once the samples were coated they were examined using a SEM (Zeiss ULTRA plus FEG SEM).

It has been established that fibrin networks, and therefore blood clots consist of thick and thin fibrin fibers [19, 20]. In order to determine whether changes in fibrin fiber thickness were present when comparing healthy and metabolic syndrome TIA individuals, 50 random fibrin fibers were measured for each participant on SEM micrographs using ImageJ (Version 1,74i, Java).

### Atomic force microscopy

#### Sample preparation

AFM was used to study the elastic properties of the platelets and fibrin fibers of the two groups. The preparation of platelets and fibrin networks for AFM is identical to that of SEM, except after drying the samples with HMDS; the samples were not coated with carbon. The samples were left to air dry before being analyzed.

### AFM measurements and imaging

Topographic images were obtained to compare the morphology of the cells and fibers studied for AFM to that of the SEM. The images were obtained by using an AFM (Dimension Icon, Bruker, USA). AFM was performed in PeakForce™ QNM™ (Quantitative Nanomechanical Property Mapping) mode. Peakforce™ QNM™ is similar to that of classic tapping where the amplitude oscillation is kept constant, but differs in that the maximum force applied by the probe was controlled [21].

### Membrane deformability (elasticity)

Deformability (elasticity) measurements were performed on the platelets and fibrin networks of the healthy individuals and patients in the experimental group to determine whether nano-mechanical property differences were present. A rapid force-distance curve was recorded at each pixel. Calibration of the cantilever’s deflection sensitivity and spring constant allowed the rapid quantitative analysis of these force-distance curves on a number of different areas on the sample. The curve obtained was used to calculate Young’s modulus and also to form adhesion images (between the slope of the curve and the minimum point of the curve). Deformation is calculated using the variation between zero and the maximum force applied. Energy dissipation can be calculated by determining the area between the approach and the development of the retract curve [22–24]. Different silicon nitride cantilevers (Bruker, USA) and settings were used for the measurement of each of the specific components investigated (See Table 2).

**Table 2:** Cantilevers and cantilever parameters (nominal values)

Component investigated	Probe name	Spring constant (N/m)	Resonant frequency	Tip radius	Peak force (nN)
Fibrin Fibers	OTESPA	12–103	345–361 kHz	15 nm	150
Platelet	ScanAsyst	0.4	70 kHz	8 nm	4

Ten cells or areas in the case of fibrin from each patient were studied. A 500 nm by 500 nm area was scanned by performing 128 by 128 data points of the individual force curve measurements. NanoScope Analysis (Version R3, Bruker, USA) was used to analyze 50 randomly selected force curves to fit to the modulus model to the unloading portion of the curve. For this study the Derjaguin-Muller-Toporov Model was used for the determination of Young’s Modulus by fitting the slope of the obtained force-distance curve to the model [25]. A curve was only incorporated into the final modulus measurements if the goodness of fit of the data curve to the modulus model was above 0.85. The goodness of fit is determined between the model and the acquired data curve by calculating the ratio of explained variation to total variation in the data set. Young’s modulus is used to gauge the rigidity of the membrane that is measured. The rigidity is representative of the elasticity or deformability of the cell membrane and is usually defined by the stress divided by the strain on the specific membrane. Higher values indicate decreased deformability, which is highly relevant to the normal functioning of red blood cells, platelets and fibrin networks.

### Thromboelastography^®^

TEG^®^ was utilized to study the hemostatic profiles of the participants. Whole blood of the participants was obtained in citrate tubes. The blood was centrifuged at 300 × g for 10 min to obtain PRP. The PRP was stored in 500 μl aliquots in a −70 °C freezer. On the day of experimentation, the aliquots were removed from the freezer and rapidly thawed at room temperature. TEG^®^ analysis of the individuals was performed on the same day to prevent variations in machine function from day to day. Two samples were run simultaneously in the 2 channels of the TEG^®^. Since it has been found that red blood cells influence the mechanical properties of clots due to their viscoelastic properties [26], PRP was used to assess the functioning of the coagulation cascade in the presence of platelets. The citrated PRP (340 μl) was added to the oscillating cup and 20 μl of calcium chloride (CaCl_2_) added to activate the coagulation process. The process was allowed to run until LY30 was reached. Anticoagulant and antiplatelet medication was not considered a reason to exclude the patient as these treatments do not affect TEG^®^ analysis, since the action of thrombin masks any antithrombotic or antiplatelet activity [27, 28].

### Statistical analysis

#### In SEM to compare fibrin fiber thickness

The measurements of each participant were done using ImageJ (Version 1,74i, Java) and the values were compared statistically. Statistical analysis was performed on Statistical Analysis System (SAS) using repeated measures analysis of variance (ANOVA). A *p*-value of less than 0.05 was considered significant.

#### In AFM to compare platelet- and fibrin fiber-elasticity

The Young’s modulus of 50 randomly selected force-distance curves with good fit on each cell or area of every patient, were compared between the 2 groups (healthy and TIA individuals). SAS software was used to perform the comparison by utilising the generalized mixed model for repeated measures function. A *p*-value of 0.05 was considered statistically significant. These comparisons were performed for platelet- and fibrin fiber-elasticity.

#### In TEG^®^ to compare hemostatic profiles of the 2 groups

The non-parametric Mann–Whitney *U* test was performed using the SAS software. A two-tailed, one sided test was performed to determine the *p*-value. A *p*-value of less than 0.05 was considered statistically significant.

## Results

### Scanning electron microscopy

SEM is a useful tool for studying morphological alterations of platelets that could be indicative of activation and fibrin fiber arrangement. Red blood cell alterations may affect the density of the clot and the degree of platelet activation, for this reason whole blood was not included in this study.

**Fig. 1:**
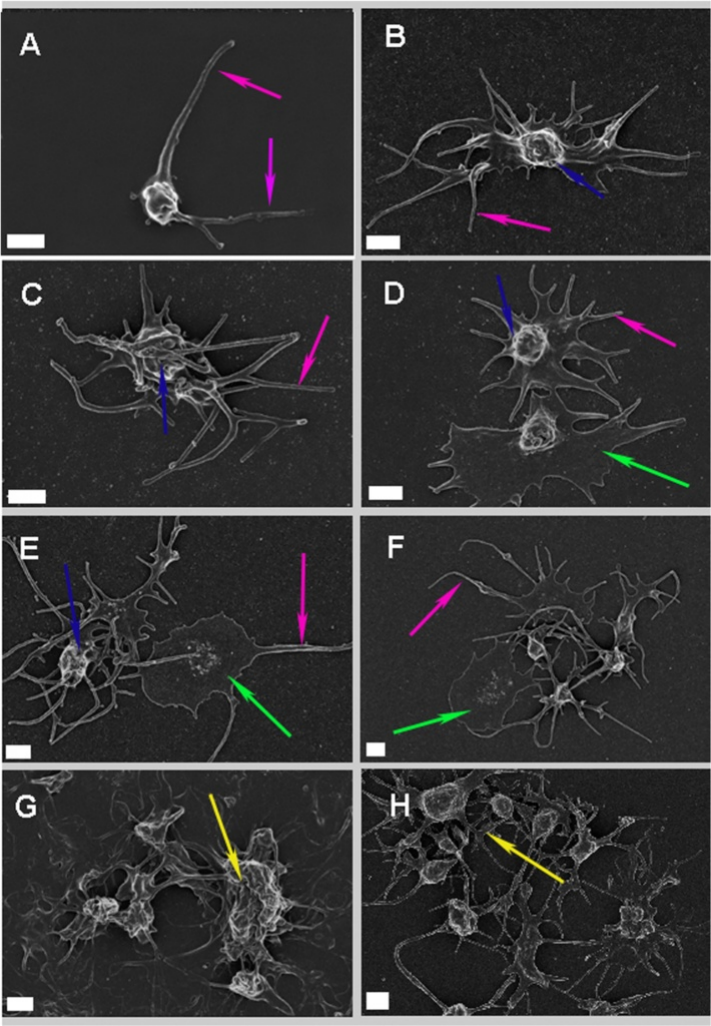
Platelets of TIA individuals in the presence of metabolic syndrome. **a**: Platelet of a typical healthy individual. **b**-**e**: TIA platelets. **f**-**h**: TIA platelet interactions and aggregates (Scale bar: 1 μm). Pink arrows: Pseudopodia. Blue arrows: OCS. Green arrows: Platelet spreading. Yellow arrows: Platelet aggregates

Figure 1 represents the platelets of healthy- and TIA-individuals. The healthy platelets are spherical with few pseudopodia visible (Fig. 1a) indicated by pink arrows. The presence of numerous pseudopodia, change in shape and spreading are indicative of activation, but is not present or expected in the healthy individuals [29]. Since these healthy individuals are not suffering from any inflammatory condition and no thrombin was added in vitro, inert or slightly activated platelets were expected. Slight activation may be due to contact activation during the preparation of the sample. The healthy platelets are consistent with previously published results [30].

Figure 1b to h represents the platelets of the TIA patients in the presence of metabolic syndrome included in this study. In Fig. 1b and c the typical platelets of the patients can be seen. These platelets are characterized by numerous pseudopodia (pink arrows) and spreading (green arrows) indicating activation. In Fig. 1d, e and f platelet-platelet interactions, also indicative of platelet activity, can be seen. Importantly, platelets wees aggregates are present (as seen in Fig. 1g and h depicted by yellow arrows) in the TIA individuals. The blue arrows in Fig. 1b, c, d and e indicate open canalicular systems (OCSs) that allow granules inside the platelet to exit the platelet.

**Fig. 2:**
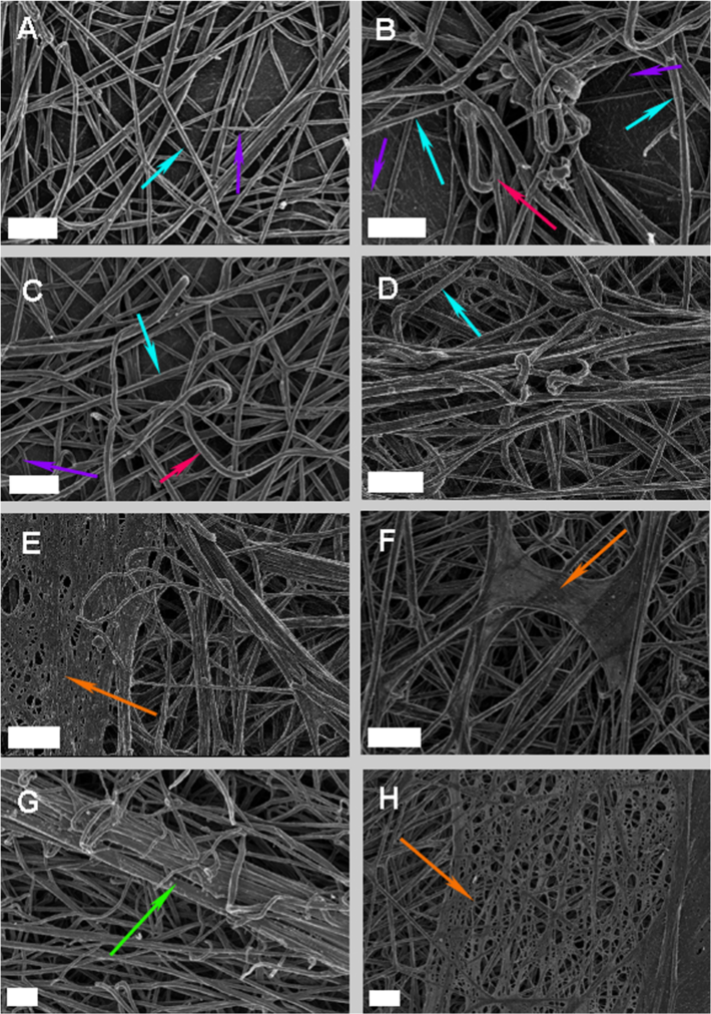
Fibrin networks of a typical healthy individual and TIA individuals. **a**: Healthy fibrin network (**b**-**h**): Fibrin network of TIA in the presence of metabolic syndrome (Scale bar: 1 μm). Turquoise arrows: Thick major fibers. Purple arrows: Thin minor fibers. Orange arrows: Net-like covering of thin fibers. Red arrows: Bending, less taut fibers. Green arrow: Sticky mass of fibrin fibers

The fibrin networks of the healthy and TIA individuals were also studied using SEM. As briefly mentioned the fibrin networks of healthy individuals typically consist of thick or major fibers (turquoise arrows) and thin or minor fibers (purple arrows) [19, 20]. The thick major fibers make up the majority of the clot as shown in Fig. 2a. In contrast the fibrin networks of TIA individuals have an altered appearance as shown in Fig. 2b to h when compared to that of control individuals (Fig. 2a). Fibrin fibers appear less organized with a higher density of thick major fibers and minor fibers forming a thick net (orange arrows) covering the clot in some individuals (Fig. 2e, f and h). Upon closer examination the fibers are also less taut, folding and twisting (red arrows) forming an irregular web of fibers as seen in Fig. 2b and c. Fibers also appear “sticky” creating thick masses of fibrin fibers (green arrow) as visualized in Fig. 2g.

Measurements of fibrin fiber thickness in the 2 groups were performed and a mean of 98.682 nm and 164.365 nm was determined in the control and experimental groups respectively. A *p*-value of <0.001 was determined indicating that a statistically significant increase in fibrin fiber thickness was present in the experimental group.

### Atomic force microscopy

**Fig. 3:**
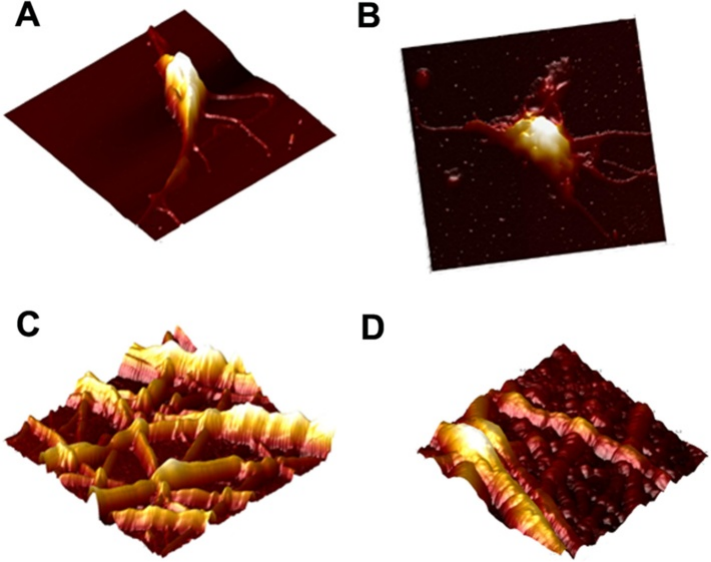
AFM topography of platelets and fibrin networks. **a**: Healthy platelet. **b**: TIA platelet. **c**: Fibrin network of healthy individual. **d**: Fibrin network of individual suffering a TIA

Due to the finding on the SEM, AFM was performed on the PRP of the individuals participating in this study to determine whether, in conjunction to the morphological changes, alterations in the nano-mechanical properties of the platelets and fibrin network fibers, that could affect coagulation, were present. Figure 3 represents the images obtained using AFM, indicating that the morphological findings on the AFM correlate with the findings on the SEM micrographs since platelet- and fibrin network-morphology appear similar with both techniques. Figure 3a represents the platelet of a healthy control individual and Fig. 3b the platelet of a TIA individual with activation visible (as seen with the presence of pseudopodia and spreading). Figure 3c and d (healthy participants) and 3d (experimental group) represent the fibrin networks of the 2 groups where Fig. 3d shows a denser structure in comparison to the healthy group, similar to that seen using SEM.

Young’s modulus, used to measure the nano-mechanical properties of the platelets and fibrin fibers were comparable to results obtained in previous studies [31]. The *p*-values obtained using SAS are represented in Table 3. Young’s modulus increased significantly (*p*-value < 0.05) in both the platelets and fibrin networks of the experimental group, indicating that the elasticity of these components decreased in the experimental participants.

**Table 3:** The *p*-values obtained from generalized mixed model for repeated measures between the healthy and experimental groups

Sample	Mean	Median	*p*-value
Control platelet	48.786	46.3	0.0042*
TIA platelet	54.587	51.1	
Control fibrin fiber	12910.84	11505	0.0007*
TIA fibrin fiber	27871.19	22263	

* Indicates statistically significant *p*-values

### Thromboelastography^®^

**Fig. 4:**
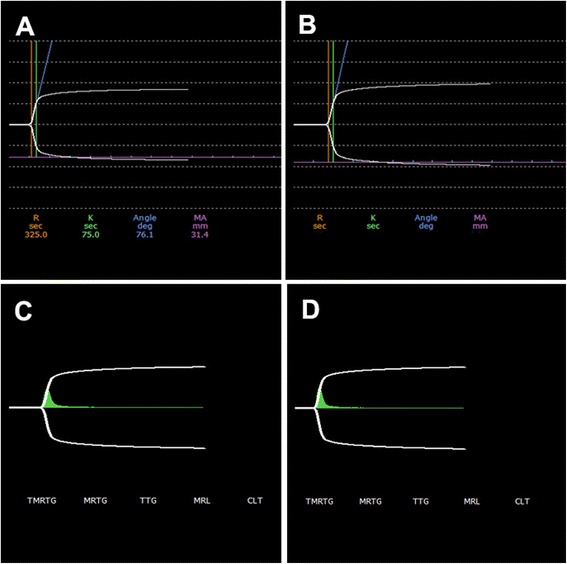
TEG^®^ trace and v-curve obtained from healthy and TIA individuals. **a**: Trace representative of healthy individuals. **b**: Trace of TIA individuals. **c**: v-curve of healthy individual. **d**: v-curve of a TIA individual

The traces obtained from the PRP of the participants are represented in Fig. 4. Figure 4 represents the trace from which reaction time (r-time), k-time, alpha angle (α-angle), maximum amplitude (MA) and clot lysis after 30 min (LY30) was determined. Figure 4a represents the traces from healthy control individuals and Fig. 4b represents the traces obtained form the experimental group. The traces appear similar in both groups regarding shape and amplitude. Figure 4c and d represent the v-curve from which time to maximum rate of thrombus generation (TMRTG), maximum rate of thrombus generation (MRTG) and total thrombus generation (TTG) were determined. Figure 4c represents the v-curve obtained from the healthy participants and Fig. 4d the v-curves from the experimental group. No differences could be observed between the participants of the two groups.

In order to determine whether the viscoelastic properties of the clots differed between the healthy and the experimental group, statistical analysis was performed on each parameter measured. The *p*-values obtained in the comparison of the healthy and TIA group are summarized in Table 4. None of the parameters showed statistically significant differences (*p*-value less than 0.05) between the two groups. This indicated that both the viscoelastic properties of the clot and the coagulation process were similar in all participants regardless of the presence of metabolic syndrome. LY30 was determined for all the individuals, but in all cases it was found to be 0 %, indicating that no clot lysis had taken place 30 min after maximum amplitude was reached in any of the participants, consistent with normal coagulation.

**Table 4:** The *p*-values of TEG^®^ parameters obtained with the Mann–Whitney *U* test

Parameter	Mean	Median	*p*-value
Control r-time	510	502.5	0.911
TIA r-time	480	522.5	
Control k-value	185	140	0.853
TIA k-value	137	132.5	
Control Alpha(α) angle	70.58	69.15	0.436
TIA Alpha(α) angle	69	68.8	
Control MA	31.62	30.25	0.190
TIA MA	37.86	35.7	
Control G	2.42	2.15	0.165
TIA G	3.32	2.8	
Control MRTGG	6.532	5.645	1.000
TIA MRTGG	6.279	6.29	
Control TMRTGG	9.749	9.29	0.796
TIA TMRTGG	9.599	10.25	
Control TGG	276.999	245.95	0.190
TIA TGG	469.401	313.725	

## Discussion

Platelets are essential in the prevention of blood loss and platelet activation is the first sign of coagulation cascade activation [11]. Platelet shape changes consistent with activation [32] was observed in the experimental group indicating that the coagulation cascade had been activated. This activation occurred spontaneously (without the artificial activation of the coagulation cascade by the addition of thrombin) in the experimental group. The same activation was not present in the experimental group indicating that the coagulation cascade had only been activated in the TIA individuals. These findings confirm the hypothesis of Wu and Hoak in 1975 as well as Hirabayashi and his colleagues in 2004 that platelet activation is closely associated with TIA [33, 34]. Since chronic inflammation is present in these individuals and inflammation is closely associated with activation of the coagulation system, it can be concluded that the activation of platelets occur prior to the cerebral event. This indicates that a procoagulant environment is present in the individuals prior to the onset of the symptoms of the TIA and therefore before formation of the pathological clot. This finding is crucial in the treatment and prevention of TIA since platelet activation is not a result of the ischemic event, but rather plays a role in the development of the attack. Platelet counts were found to be normal in the diseased individuals indicating that the number of platelets did not contribute to the attack, but rather that the activation of the platelets was the crucial step prior to the formation of the clot.

It is well known that platelets increase clot elasticity in normal physiology since they make up approximately 80 % of a thrombus; consequently any alterations in the membrane characteristics (such as elasticity) of the platelets will affect the formed thrombus as well [35]. In the presence of chronic inflammation (as seen in this study) the elasticity of the platelets decreased significantly, giving rise to more rigid platelets with a decreased ability to contract during the final stages of clot formation. A decrease in clot retraction, due to decreased platelet contraction, could consequently result in less taut fibers and less organized fibrin networks.

Together with platelets, fibrin fibers make up the majority of the structure of the clot and are the main determinants of the clot mechanics. The fibrin networks of the diseased participants were therefore studied to determine whether alterations in fibrin networks could contribute to changes in clot formation that could lead to the development of a TIA. The fibrin networks of the TIA individuals appeared greatly altered when compared to healthy individuals. These findings are consistent with the findings of other studies that abnormal coagulation plays a major role in ischemic events [36]. The networks appeared denser, less organized with sticky thick fibers that clump together, most likely due to a favouring of lateral aggregation of the protofibrils, this is also consistent with other studies where it was found that denser fibrin networks were associated with a reduction in clot permeability and consequently clot lysis [36–38]. The presence of inflammatory cytokines could further decrease the permeability of the clot as found by Pera et al. in 2015 [39, 40]. Although LY30 measured on the TEG^®^ did not indicate hyperfibrinolysis it cannot comment on a decrease in clot fibrinolysis which could be present due to altered fibrin network formation. In some areas the thin fibers form a net that covers large parts of the formed clot. The fibrin fibers of the TIA individuals are significantly thicker than those of the healthy individuals, as seen with statistical analysis. This increase in thickness is most likely due to the propensity of the fibers to stick together as seen in the experimental group. The maximum amplitude (MA) of the clot measured using TEG^®^ corresponds to the speed of fibrin build-up and fibrin cross-linking during clot formation and could therefore be used to study whether the altered fibrin networks affected clot formation. The MA values of the TIA individuals were similar to that of the healthy group. It can therefore be concluded that the formation of the clot was not altered, however the MA does not comment on the quality or thickness of the fibers that could contribute to the formation of the pathological thrombus in the TIA event. Similar to the elasticity of the platelets, the elasticity of the fibrin fibers also decreased (seen with the AFM results) substantiating the theory that a decrease in platelet elasticity will result in a decrease in fibrin fiber elasticity, since clots containing platelets are more elastic than clots without platelets. Less elastic fibers will make it even more difficult for clot retraction to take place explaining the slackness of the fibers seen in the TIA individuals using SEM and AFM. Although fibrinogen concentration may contribute to the formation of a pathological clot, this is probably not the case here, since the rate of clot formation (as measured by TEG^®^ and several plasma based tests) was not significantly different from the healthy controls as would be expected in increased fibrinogen concentrations. Since chronic inflammation is present in the diseased individuals and inflammation activates the coagulation cascade, the changes in the fibrin networks can most likely also be attributed to the continuous inflammatory response that activates platelets. This activation of the platelets, since they function very closely with the fibrin network, is most likely the initiation step of the fibrin network changes seen in the experimental group.

No changes in any of the parameter measured with TEG^®^ could be detected between the healthy control group and the diseased experimental group, indicating that the overall hemostatic profile of the individuals were not affected by metabolic syndrome leading to TIA and that the viscoelastic properties of the clots are similar in the healthy and diseased populations.

**Fig. 5:**
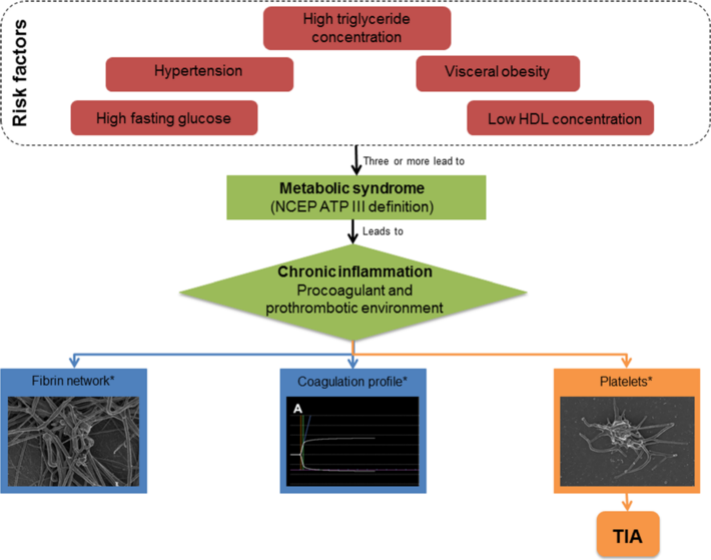
Figure summarizing the findings of this manuscript. *Findings represented in Table 5. HDL: High-density lipoprotein; TIA: Transient ischemic attack; NCEP ATP III: National cholesterol education program – Adult treatment panel III. Since it is hypothesised that metabolic syndrome causes chronic inflammation that unnecessarily activates the platelets and it is believed that the platelet alterations cause the changes seen in the fibrin networks, this arrow indicates the major culprit in the development of TIA

Figure 5 and Table 5 represent the findings of this manuscript and how it relates to the development of TIA.

**Table 5:** Summary of the findings of this manuscript according to techniques used

Technique	Platelets	Fibrin networks	Conclusions drawn
SEM	Platelet activation present seen with presence of pseudopodia and spreading	Denser, less organized fibrin networks in experimental group	Platelet activation present, altered fibrin network formation with the addition of thrombin affecting clot characteristics and possibly the hemostatic process
	Platelet aggregation present in the experimental group	Thick major fibers sticky and clumped together and thicker than fibers in healthy individuals	These alterations may be due to changes in elasticity
		Fibers making up clots of the experimental group were less taut with visible fibrin fiber ends not found in the healthy group	
AFM	Platelets are less elastic in the diseased individuals	Fibrin fibers are less elastic in the experimental group	A decrease in platelet elasticity affects platelet contraction and therefore clot retraction, resulting in altered fibrin network elasticity and network morphology
TEG^®^	N/A	N/A	TEG^®^ was used to determine whether the alterations obtained with the results of the other techniques affected the hemostatic profile of the individuals, but was found not affect the overall coagulation process

## Conclusion

The findings of this study substantiates the results of other publications which indicate that fibrin network morphology is altered in ischemic events such as stroke [38]. Furthermore it has been postulated that fibrin formation occurs faster in ischemic events in comparison to normal physiology, producing a denser clot (seen with SEM) with increased resistance to fibrinolysis, this study suggests that the quicker fibrin formation and altered fibrin clot properties is a result of the premature activation of the coagulation cascade by platelets that are constantly activated in the presence of chronic inflammation as is the case in metabolic syndrome. This is further shown by the findings of the TEG^®^ indicating that the coagulation cascade is not altered in the experimental group showing that the cause of the formation of the clot is due to alterations in the hemostatic profile of the participants. Platelet- and fibrin fiber-elasticity is decreased resulting in a less permeable clot. Although fibrinolysis may be impeded by the alterations found in the components of coagulation found in this manuscript, fibrinolysis will still occur, leading to the transient period of the symptoms that characterize a TIA.

The findings of this thesis contributes to research and the clinical setting by showing that treatment of a TIA and prevention of future TIA or stroke should focus on the prevention of platelet activation that could prevent to the formation of an abnormal fibrin network and a pathological thrombus.

A limitation to this study is the number of participants; however, this study is adequately powered.

### Ethics, consent and permissions

Ethics approval was obtained from the University of Pretoria Human Ethics Committee. Ethics number: 237/2012 with amendment in 2015, new number 24/2015.

### Consent to publish

Written consent to use data and to publish was obtained form each individual participating in this study.

## Abbreviations

AFM: Atomic Force Microscopy
ANOVA: Analysis of Variance
ECG: Electrocardiogram
HDL: High density lipoprotein
HIV: Human Immunodeficiency Virus
HMDS: Hexamethyldisilazane
LY30: Clot lysis 30 min after MA
MA: Maximum Amplitude
MRTG: Maximum Rate of Thrombus Generation
NCEP ATP III: National Cholesterol Education Program, Adult Treatment Panel III
OCS: Open Canalicular System
OsO_4_: Osmium Tetroxide
PB: Phosphate Buffer
PRP: Platelet-rich Plasma
QNM™: Quantitative Nanomechanical Property Mapping™
r-time: Reaction Time
SANBS: South African National Blood Service
SAS: Statistical Analysis System
SEM: Scanning Electron Microscopy
TEG^®^: Thromboelastography^®^
TMRTG: Time to Maximum Rate of Thrombus Generation
TIA: Transient Ischemic Attack
TTG: Total Thrombus Generation
